# Mode of Proximal Tubule Damage: Differential Cause for the Release of TFF3?

**DOI:** 10.3389/fimmu.2016.00122

**Published:** 2016-03-30

**Authors:** Zinah Zwaini, Dalia Alammari, Simon Byrne, Cordula Stover

**Affiliations:** ^1^Department of Infection, Immunity and Inflammation, College of Medicine, Biological Sciences and Psychology, University of Leicester, Leicester, UK; ^2^Kufa College of Medicine, Najaf, Iraq

**Keywords:** trefoil factor 3, nutrient starvation, hypoxia, renal, protein-induced damage

## Abstract

Proximal tubular epithelial cells are particularly sensitive to damage. In search of a biomarker, this study evaluated the potential of different cell activation models (hypoxia/replenishment and protein overload) to lead to a release of trefoil factor 3 (TFF3). Surprisingly, we found disparity in the ability of the different stimuli to enhance the intracellular abundance of TFF3 and its release: while conditions of nutrient starvation and damage associated with replenishment lead to intracellular abundance of TFF3 in the absence of TFF3 release, stimulation with an excess amount of albumin did not yield accumulation of TFF3. By contrast, incubation of cells with a purified λ light chain preparation from a patient with multiple myeloma provoked the presence of TFF3 in the cell supernatant. We, therefore, propose that elevations of TFF3 in renal disease might be more revelatory for the cause of restitution than previously thought.

## Introduction

Trefoil factor 3 (TFF3) has a role in restitution (cell migration to heal superficial lesions) and regeneration (differentiation and proliferation as repair) of epithelia. Contrasting with genes that encode other members of the TFF peptide family, TFF3 mRNA is expressed in the cortex of the kidney (1). TFF3 peptide was readily detectable in urine from patients with nephrolithiasis compared to normal urine (1). Elevated levels were also found in urine from patients with incident chronic kidney disease as part of a nested study with a median follow up time of nearly 9 years (2) as well as in serum of patients with chronic kidney disease stages 1–5 (3). Possible triggers for the release of TFF3 may include damage or inflammation (2).

We have developed *in vitro* models that target cells of the proximal tubular epithelial cell line HK-2 in different ways: a phase of hypoxia/nutrient starvation (HNS) and subsequent replenishment with medium models the injury observed in ischemia reperfusion; stimulation of HK-2 cells with immunoglobulin light chains (LCs) or albumin devoid of fatty acids models effects of monoclonal gammopathy and elevated protein, respectively.

The aim of this study was to compare and contrast TFF3 release in response to these experimental conditions.

## Materials and Methods

For the HNS–replenishment (HNSR) model, phenolphthalein containing growth medium [DMEM:F-12 (Thermo Fisher Scientific Ltd.) with l-glutamine (200 mM), 10% (v/v) fetal calf serum (FCS), 200 μg/ml recombinant human epidermal growth factor (Sigma-Aldrich Company Ltd., Dorset, UK), penicillin (100 IU/ml), and streptomycin (100 μg/ml)] was replaced with serum-free glucose-free Locke’s buffer (154 mM NaCl, 5.6 mM KCl, 2.3 mM CaCl_2_, 1 mM MgCl_2_, 3.6 mM NaHCO_3_, and 5 mM HEPES, pH 7.2) and proximal tubular epithelial cells of normal human kidney (HK-2) (ATCC CRL-2190) were exposed to a customized gas mixture (0.5% O_2_, 5% CO_2_, 94.5%N_2_; BOC Ltd., Guildford, UK) in a custom-made chamber (S. Byrne and University of Leicester workshop). After 6 h at 37°C, the cells were replenished with growth medium and returned to 5% CO_2_ for 24 and 48 h (37°C), respectively.

For the protein stimulation model, HK-2 cells were grown in phenolphthalein containing DMEM:F-12 supplemented with l-glutamine, 15 mM HEPES, 10% (v/v) FCS, recombinant human epidermal growth factor (200 μg/ml), insulin/transferrin/sodium selenite (ITS), triiodothyronine (4 pg/ml), and hydrocortisone (100 μg/ml). The cells were sub-cultured in six-well plates for 24 h. Then, medium was changed to serum-free medium with 5 mg/ml human serum albumin essentially free of fatty acids (FAF-HSA) (Sigma) diluted in endotoxin-free water or λ LCs purified by chromatography from the urine of a patient with multiple myeloma. The efficiency of endotoxin removal from the preparation by High-Capacity Endotoxin Removal Spin Column (Thermo Fisher Scientific) was confirmed by chromogenic Limulus amebocyte lysate assay kit (Pierce). Cells were stimulated for 24 and 72 h. A tubulotoxic effect of fatty acid carrying albumin has previously been shown *in vitro* at 24 h of stimulation using primary human proximal tubular cells (4). Newman et al. used human serum albumin 95% pure and free of fatty acid for their stimulation experiments in concentrations from 0.05 to 5 mg/ml to cover the range of the normal and the pathological condition in kidneys (5). Five grams per liter were the concentration of λ LC recorded for the multiple myeloma patient’s 24-h urine.

Protein lysates (200 μg) were prepared from both experiments and analyzed by R&D Systems Proteome Profiler™ Human Kidney Biomarker Array Kit. Image J 1.49 software was used to quantify intensities on developed X-ray film. For Western blotting, rabbit anti HIF-1α (1:500, Biorbyt) and mouse anti β-actin (clone AC-74, 1:5000, Sigma) were used as primary antibodies. HRP-conjugated swine anti-rabbit immunoglobulins (1:3000, DAKO) and goat anti-mouse immunoglobulins (1:2000, DAKO) were used for detection with ECL reagents (Pierce). TFF3 antibody was purchased from Biorbyt Ltd. (Cambridge, UK) and used at 1:500 with DAKOmate Envision detection kit (1:40). V.5 Image Lab software (BioRad) was used to quantify reactivities on developed X-ray films. TFF3 was measured by R&D Systems™ Quantikine^®^ ELISA human TFF3 immunoassay (Minneapolis, MN, USA), and levels were calculated using a linear standard curve. Undiluted supernatants were used.

## Results and Discussion

**Figure 1:**
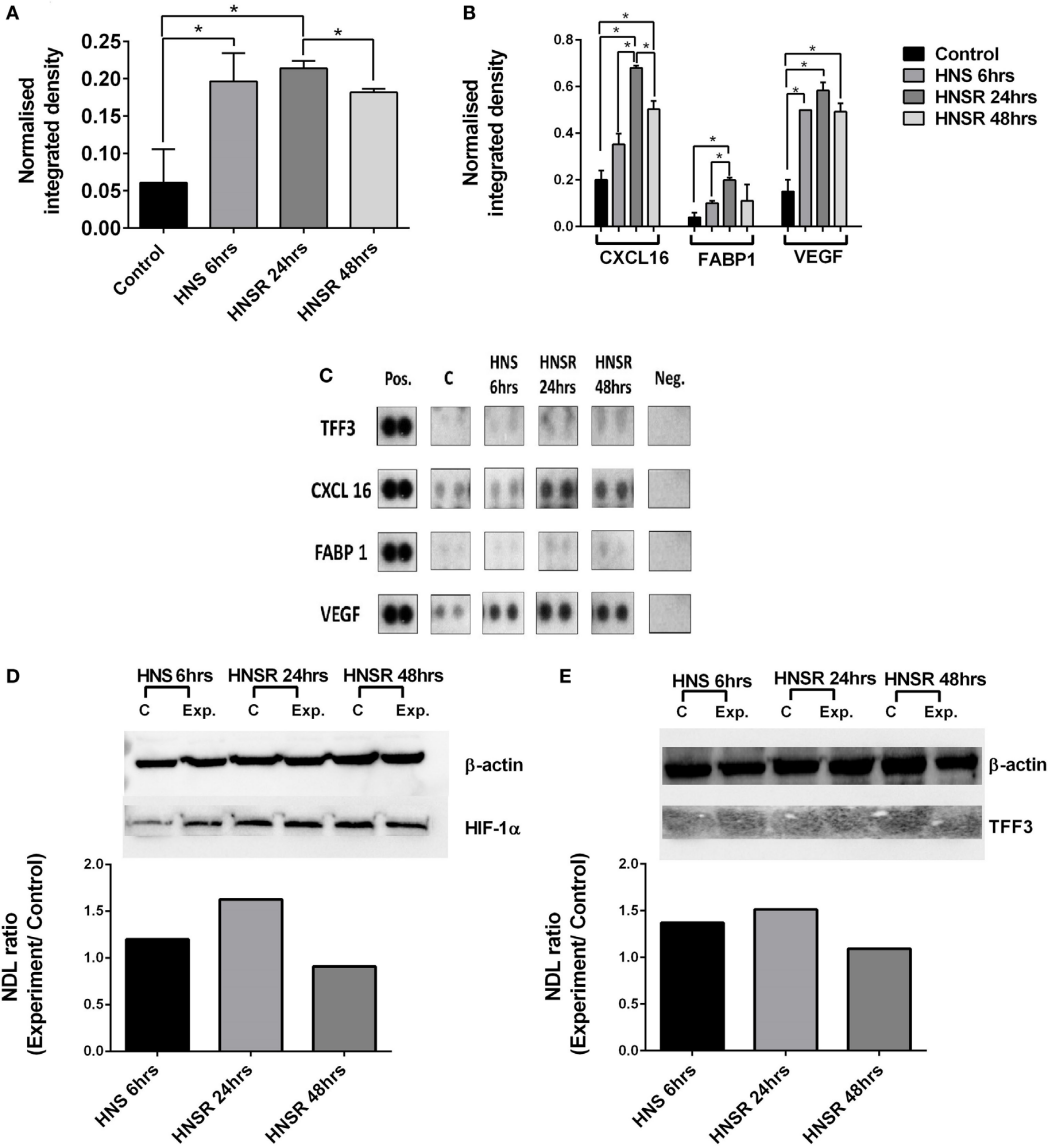
**Densitometric analysis of proteome array reactivities for TFF3 (A) and characteristic markers of hypoxia (FABP1, VEGF) and inflammation (CXCL16) (B) using cell lysates from HK-2 cells exposed to hypoxia/nutrient starvation (HNS) and replenishment (HNSR) for 24 and 48 h**. Densities obtained are shown in **(C)**. These were normalized to a reference (pos) and the background was subtracted. Data are presented as mean ± SD and were compared using Student’s multiple *t*-test. *p* < 0.05 was considered statistically significant. GraphPad Prism 6.07 was used for statistical analysis (GraphPad Software, La Jolla, CA, USA). HIF-1α abundance is increased at the early replenishment time point when normalized to a housekeeping protein β-actin and compared to the normoxic control **(D)**. Normalized abundance of TFF3 (7 kDa) in cell lysates from HK-2 cells exposed to hypoxia/nutrient starvation (HNS) and replenishment for 24 and 48 h **(E)**. NDL, normalized density to loading control (Western blot).

Nutrient starvation and hypoxia were sufficient triggers for increased TFF3 abundance in lysates from HK-2 cells, and this was maintained during the replenishment phase at 24 and 48 h (Figures 1A,C). In this acute phase (24 h), effectors of hypoxia-induction (VEGF, FABP1, CXCL16) were greatly increased compared to control cells (Figures 1B,C). HIF-1α was elevated at this time point (Figure 1D). Transcription of VEGF, HIF-1, and TFF3 mRNA is induced by hypoxia *in vitro* (6, 7), *CXCL6* gene promoter has a hypoxia-sensitive response element (8), and FABP has been described as a marker of hypoxic renal cell damage (9, 10). CXCL16 and VEGF remained elevated compared to the control at 48-h medium replenishment. A commercial ELISA did not detect TFF3 in the cell supernatant, therefore, TFF3 was deemed to be below the detection limit of 39 pg/ml. This contrasts with the abundance of TFF3 found in HK-2 cell lysates: Western blot analysis of cell lysates confirmed the presence of TFF3 in lysates (Figure 1E), implying the detection of an intracellular form of TFF3 (11). While it is known that TFF3 is regulated by HIF-1α (12), it is not known how hypoxic damage or the temporary withdrawal of insulin (13) might alter secretion of TFF3.

**Figure 2:**
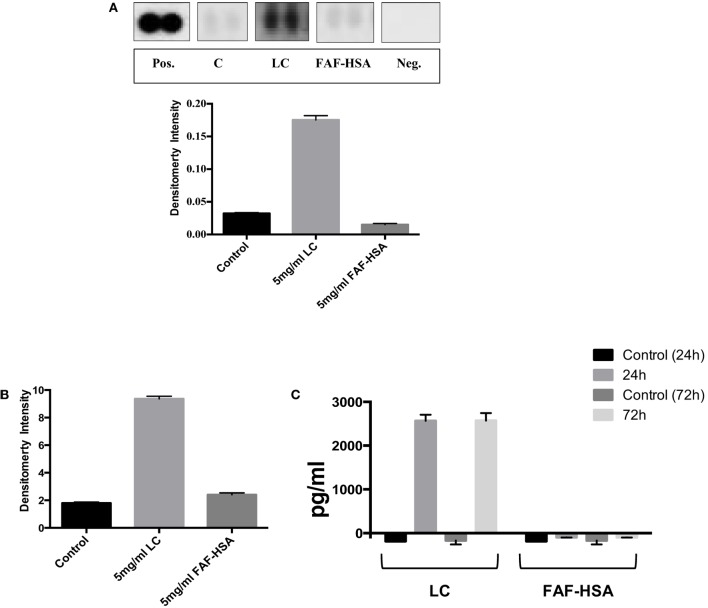
**The human kidney biomarker array (R&D Systems) was used to analyze TFF3 abundance in lysates from cells exposed to immunoglobulin λ light chain (LC) and fatty acid free human serum albumin (FAF-HSA) for 72 h in comparison with control cells (A)**. Densities were normalized to a reference (pos), background subtracted and are expressed as ±SD. **(B)** Abundance of TFF3 (in relation to β-actin) in cell lysates from HK-2 cells exposed 5 mg/ml immunoglobulin λ light chain (LC) or fatty acid-free human serum albumin (FAF-HSA) for 72 h (Western blot). **(C)** ELISA of TFF3 in the supernatants of HK-2 stimulated with 5 mg/ml LC and FAF-HSA. Untreated cells were used as control. Two independent experiments for the distinct stimulations for 24 and 72 h are shown.

By contrast, when analyzing in parallel HK-2 cells exposed to pathological concentrations of endotoxin free protein (immunoglobulin λ LC or human serum albumin, devoid of bound fatty acids), VEGF, FABP1, and CXCL16 were not increased at the 72 h time point of this *in vitro* model compared to the unstimulated control cells (data not shown). TFF3 abundance, however, was markedly elevated in those lysates obtained from cells exposed to the λ LC preparation, but not in lysates from HK-2 cells exposed to the same concentration of albumin (Figure 2A). Western blot analysis of cell lysates validated this observation: λ LC-stimulated cells showed markedly elevated TFF3 abundance, contrasting with untreated and human serum albumin-stimulated cells (Figure 2B). A commercial ELISA quantified TFF3 in supernatants of another identical experiment: nanogram amounts of the peptide were detectable from λ LC-stimulated HK-2 cells, whereas albumin-stimulated HK-2 cells were negative (Figure 2C).

We conclude that immunoglobulin LCs initiate more restitution in proximal tubular epithelial cells *in vitro* than albumin. Contrary to benign monoclonal gammopathy, which is not necessarily kidney damaging, monoclonal gammopathy of renal significance is not subclinical, and requires aggressive treatment (14). Amino acid changes in LC sequences may confer considerable toxicity to proximal tubular epithelial cells (15). In these cases, longitudinal measurements of TFF3 could be warranted.

## Author Contributions

ZZ, DA, SB, and CS: substantial contributions to the conception or design of the work; or the acquisition, analysis, or interpretation of data for the work; ZZ, DA, SB, and CS: drafting the work or revising it critically for important intellectual content; ZZ, DA, SB, and CS: final approval of the version to be published; and ZZ, DA, SB, and CS: agreement to be accountable for all aspects of the work in ensuring that questions related to the accuracy or integrity of any part of the work are appropriately investigated and resolved.

## Conflict of Interest Statement

The authors declare that the research was conducted in the absence of any commercial or financial relationships that could be construed as a potential conflict of interest.
